# Construction and verification of 5-year survival prediction model for post-op ESCC patients

**DOI:** 10.3389/fonc.2026.1798175

**Published:** 2026-04-29

**Authors:** Zeyu Wang, Chaowei Li, Chao Ren, Shuyi Li, Shasha Yuan, Xu Yang, Jing Wang, Heng Cao, Jianwei Cao, Jin Xia

**Affiliations:** 1 Department of Medical Oncology, Anyang Tumor Hospital, The Affiliated Anyang Tumor Hospital of Henan University of Science and Technology, Anyang, China; 2 Department of Thoracic Surgery, Anyang Tumor Hospital, The Affiliated Anyang Tumor Hospital of Henan University of Science and Technology, Anyang, China

**Keywords:** 5-year postoperative survival rate, esophageal squamous cell carcinoma, nomogram model, overall survival (OS), prediction model

## Abstract

**Objective:**

To develop a predictive model for the 5-year postoperative survival rate of patients with esophageal squamous cell carcinoma (ESCC), and to provide a basis for the accurate assessment of clinical prognosis.

**Methods:**

A total of 260 ESCC patients who underwent surgery between January 2017 and June 2019 were included and randomly divided into a training set (n=182) and a validation set (n=78) at a 7:3 ratio. Univariate and multivariate Cox proportional hazards regression analyses were performed in the training set to identify independent predictors of 5−year overall survival (OS). A nomogram was constructed based on the selected factors. The model’s performance was evaluated using the concordance index (C−index), calibration curves, time−dependent receiver operating characteristic (ROC) curves, and decision curve analysis (DCA).

**Results:**

The 5−year OS rate in the training set was 63.7% (116/182). Multivariate Cox regression identified five independent predictors of 5−year OS: American Joint Committee on Cancer stage (HR = 1.347, 95% CI: 1.209−1.510), lymph node metastasis (HR = 4.698, 95% CI: 3.282−8.994), tumor differentiation grade (HR = 1.235, 95% CI: 1.105−1.276), microvessel density (HR = 1.089, 95% CI: 1.023−1.159), and Vascular Endothelial Growth Factor (VEGF) expression (HR = 2.451, 95% CI: 1.127−6.372). The nomogram demonstrated good discrimination, with C−index values of 0.886 (training set) and 0.845 (validation set). The areas under the ROC curves were 0.889 (95% CI: 0.822−0.955) and 0.844 (95% CI: 0.695−0.992), respectively. Calibration curves and DCA indicated satisfactory agreement and clinical net benefit across a wide threshold probability range.

**Conclusion:**

The nomogram integrating clinicopathological and molecular factors effectively predicts 5−year OS in ESCC patients after surgery, offering a reliable visual tool for individualized risk stratification and clinical decision−making.

## Introduction

Esophageal squamous cell carcinoma (ESCC) is one of the most common malignancies globally, and its incidence and mortality rates rank among the top in digestive system tumors (1). Surgical resection is the primary treatment for early - stage ESCC, but there are still significant differences in the 5-year survival rate after surgery, and approximately 30% - 50% of patients experience recurrence or metastasis (2). Therefore, accurately predicting postoperative prognosis and formulating personalized treatment strategies have become hot topics in clinical research. Previous studies have indicated that factors such as Microvessel Density (MVD), Vascular Endothelial Growth Factor (VEGF), and clinicopathological features [e.g., American Joint Committee on Cancer (AJCC) Staging] are closely associated with the postoperative survival of patients with ESCC (3–5). However, a single indicator is difficult to comprehensively reflect the prognostic risk of patients, and there is an urgent need to integrate multi - dimensional indicators to construct an accurate prediction model. The nomogram model integrates multiple independent risk factors in a visual way and can provide a direct quantitative prognostic evaluation tool for clinicians. This study aims to establish a predictive model for the 5-year postoperative survival rate of patients with esophageal squamous cell carcinoma (ESCC) and validate its effectiveness, so as to provide a scientific basis for clinical decision-making.

## Materials and methods

### Study population

Patients with ESCC who underwent surgical treatment in our hospital from January 2017 to June 2019 were selected as the research subjects. Inclusion criteria: Pathological histology confirmed ESCC; First-time radical surgical resection; AJCC stage was I-III; Complete clinical data, including postoperative pathological reports, follow-up records, etc. Exclusion criteria: Co-existing with other malignant tumors; Receiving radiotherapy, chemotherapy or targeted therapy before surgery; Severe insufficiency of cardiac, pulmonary, hepatic or renal function; Lost to follow-up or incomplete follow-up data.

### Sample size calculation

First, sample size calculation was performed in this study: Based on the expected incidence of the adverse outcome (death within 5 years) of the primary outcome measure (5-year overall survival rate of postoperative ESCC patients) — set at 30%-40% (consistent with the actual incidence, as determined by previous literature, pilot studies, and characteristics of the target population) — a protocol matching the core statistical method (multivariate COX regression) was adopted. The calculation was completed using the ‘Sample Size Calculation’ module of SPSS 26.0, and verified using the ‘pwr’ package in R 4.2.1. With the significance level set at *α* = 0.05 (two-tailed) and the test power at 1-*β* = 80%, and a 10% loss-to-follow-up rate taken into account, the minimum required sample size was finally determined to be 152 cases. Accordingly, 260 ESCC patients with complete clinical, pathological, follow-up and molecular detection data from our hospital were enrolled, and some patients who met basic inclusion criteria were excluded due to incomplete data or loss of follow-up.

### Treatment methods

All patients underwent radical resection of esophageal cancer. The surgical approaches included transthoracic esophagectomy (Ivor-Lewis procedure) or transabdominal esophagectomy (Sweet procedure). The specific surgical method was determined based on factors such as the tumor location and the patients’ physical conditions. After surgery, according to the pathological stage and the patients’ wishes, some patients received adjuvant chemoradiotherapy. The chemotherapy regimen was platinum - based (e.g., cisplatin + fluorouracil), and the radiotherapy dose was 45–50 Gy/25–28 fractions. During the study period (January 2017 to June 2019), there were no significant changes in the surgical techniques and adjuvant therapy protocols for ESCC in our institution, which ensured the consistency of clinical treatment and excluded the potential impact of treatment protocol changes on the survival outcomes of patients.

### Data collection

General information of patients (age, gender, preoperative weight loss), clinicopathological characteristics (tumor diameter, AJCC staging, lymph node metastasis, tumor differentiation degree), molecular biological indicators (MVD, VEGF expression) and treatment - related information (post - operative adjuvant radiotherapy and chemotherapy) were collected. MVD was detected by CD34 immunohistochemical staining according to the internationally recognized gold standard method proposed by Weidner et al. (6). The number of microvessels in three high - power fields (HPF, ×200) in the tumor hot - spot area was counted, and the average value was taken. VEGF expression was detected by immunohistochemical method and was classified into high expression and low expression according to the proportion of positive cells and staining intensity.

### Follow-up and primary outcome

Postoperative follow-up time started from the date of surgery until December 31, 2024, or until the patient’s death. For patients lost to follow-up, survival data were censored at the date of last contact. Follow-up content, including outpatient re-examinations and telephone follow-ups, was conducted once every three months for two years, and then once every six months. Overall survival (OS) time was defined as the time from surgery to death or the end of follow-up, and the 5-year postoperative survival rate was set as the primary outcome.

### Statistical analysis

Data analysis was performed using SPSS 26.0 and R software. Measurement data were expressed as mean ± standard deviation, and independent - sample t - test was used for comparison between groups. Count data were expressed as the number of cases (%), and the χ² test or Fisher’s exact probability method was used for comparison between groups. Univariate and multivariate Cox proportional hazards regression analysis were performed to identify potential predictive factors associated with the primary outcome, and their hazards ratios (HR) and 95% confidence intervals (CI) were calculated. A Nomogram model was constructed based on the independent risk factors using the “rms” package in R software. Model discrimination was assessed using the concordance index (C-index) and time-dependent receiver operating characteristic (ROC) curves with calculation of the area under the curve (AUC). Calibration, which measures the agreement between predicted probabilities and observed outcomes, was evaluated using calibration curves generated via the bootstrap method with 1000 resamples. Finally, the clinical utility and net benefit of the nomogram across different threshold probabilities were assessed using decision curve analysis (DCA). A two-sided P-value<0.05 was considered statistically significant for all analyses. A total of 260 patients with ESCC were included and randomly divided into a training set (n=182) and a validation set (n=78) at a ratio of 7:3, which is a widely accepted and guideline-recommended splitting strategy in clinical prediction model construction to ensure sufficient sample size for model fitting and effective validation (7).

## Results

### Comparison of baseline data of patients in the training set and the validation set

A total of 260 patients with ESCC were included and randomly divided into a training set (n=182) and a validation set (n=78) at a ratio of 7:3. In the training set, there were 66 death events and 116 patients who survived, resulting in a 5-year overall survival (OS) rate of 63.7% (116/182). No statistically significant differences were observed in any of the variables between the training and validation sets (all *P* > 0.05), confirming that the dataset division was balanced and comparable (**Table 1**).

**Table 1:** Comparison of patient data between the training set and the validation set.

Factors		Training set (n=182)	Validation set (n=78)	χ²/*t*	*P*
Age (years)		66.89 ± 9.01	66.44 ± 9.02	0.369	0.713
Sex	Male	104(57.02)	46(58.82)	0.075	0.784
	Female	78(42.98)	32(41.18)		
Tumor diameter (CM)		4.12 ± 0.95	4.09 ± 0.89	0.238	0.812
AJCC stage	I/II	129(71.07)	54(68.63)	0.071	0.790
	III	53(28.93)	24(31.37)		
Lymph node metastasis status	Yes	89(48.76)	38(49.02)	0.001	0.980
	No	93(51.24)	40(50.98)		
Tumor differentiation degree	High	75(41.32)	31(39.22)	0.050	0.826
	Moderate-low	107(58.68)	47(60.78)		
MVD(number/HPF)		34.86 ± 5.23	33.67 ± 4.98	1.705	0.089
VEGF	High expression	74(40.50)	35(45.10)	0.400	0.528
	Low expression	108(59.50)	43(54.90)		
Pre-operative weight loss	Yes	87(47.93)	38(49.02)	0.018	0.892
	No	95(52.07)	40(50.98)		
Post-operative adjuvant radiotherapy and chemotherapy	Yes	81(44.63)	31(39.22)	0.505	0.477
	No	101(55.37)	47(60.78)		

### Survival curve analysis

**Figure 1:**
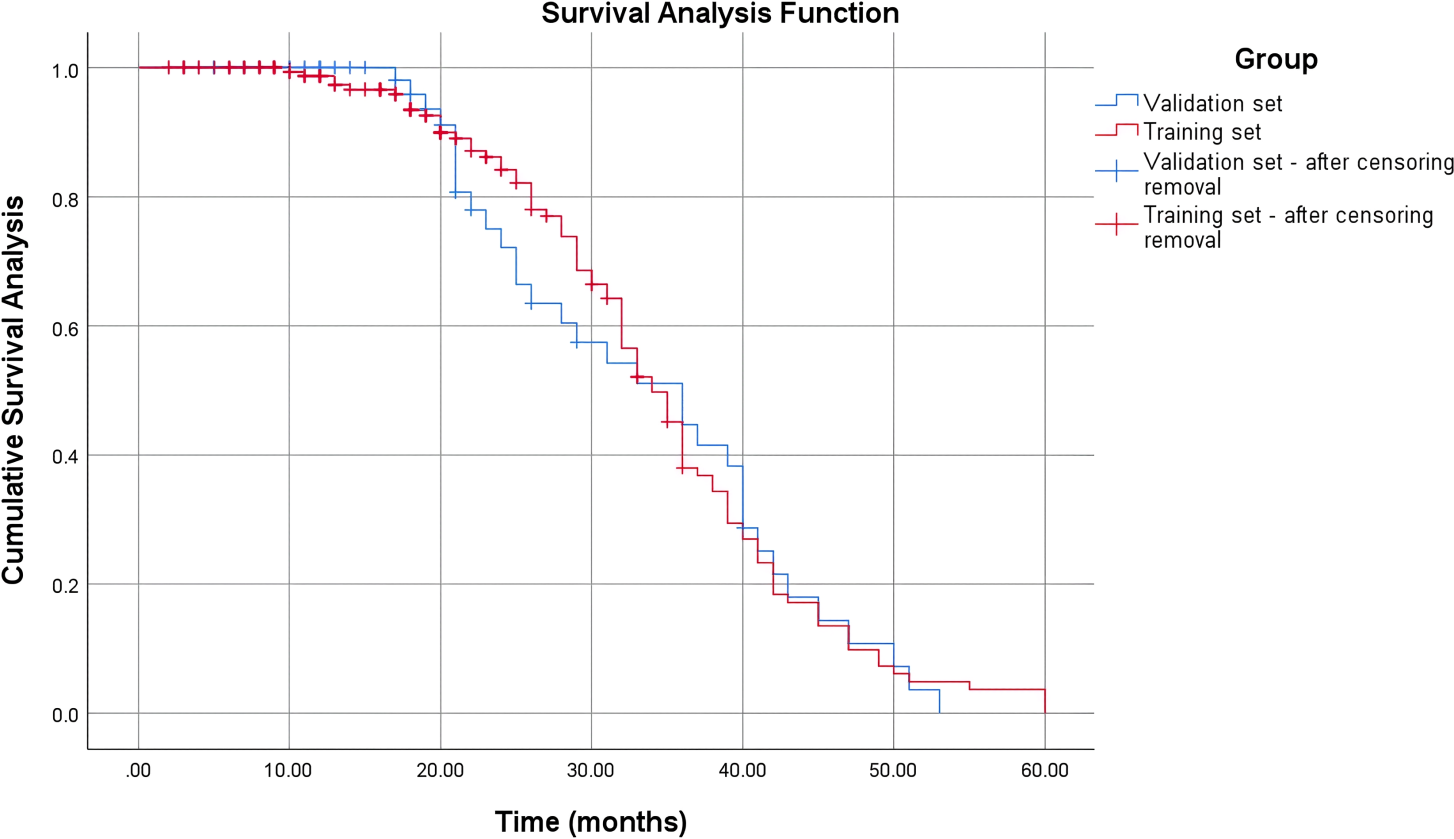
Kaplan-Meier survival curves for 5-year overall survival in the training and validation sets. “Train” represents the training set; “Validation” represents the validation set; ns indicates no statistically significant difference (P>0.05).

To further verify the balance of baseline survival outcomes between the two sets, Kaplan-Meier curves for 5-year overall survival were plotted for the training and validation sets (**Figure 1**). The curve trends showed that the survival probabilities over time were highly consistent between the two sets. The log-rank test indicated no statistically significant difference (*P*>0.05), confirming that the random grouping did not introduce survival bias and provided a reliable foundation for subsequent model construction and validation.

### Univariate and multivariate Cox regression analysis

The variable assignments for the regression analysis were detailed in **Supplementary Table 1**. Univariate Cox proportional hazards regression analysis was performed to identify variables associated with 5-year OS in ESCC patients. The results indicated that tumor diameter, AJCC stage (III vs. I/II), lymph node metastasis (Yes vs. No), tumor differentiation grade (High vs. Moderate-Low), MVD (per 1-number increase), VEGF expression (High vs. Low), preoperative weight loss (Yes vs. No), and postoperative adjuvant therapy (Yes vs. No) were significantly associated with 5-year OS (all *P* < 0.05).

Multivariate Cox regression analysis further identified five independent predictors of 5-year OS after excluding non-independent factors to reduce model redundancy: AJCC stage (III vs. I/II) (HR = 1.347, 95% CI: 1.209-1.510), lymph node metastasis (Yes vs. No) (HR = 2.915, 95% CI: 1.982–4.288), tumor differentiation grade (High vs. Moderate-Low) (HR = 1.235, 95% CI: 1.105-1.276), MVD (per 1-number increase) (HR = 1.089, 95% CI: 1.023-1.159), and VEGF expression (High vs. Low) (HR = 2.451, 95% CI: 1.127-6.372) (all *P* < 0.05) (**Table 2**). Multicollinearity among the core variables (AJCC stage, MVD, and VEGF) was assessed using variance inflation factor (VIF), and all VIF values were below 3, indicating no significant multicollinearity that would adversely affect the model. This study further conducted multivariate COX regression analysis on the validation set to assess the stability of the independent predictors identified in the training set. The results showed that AJCC stage, lymph node metastasis, tumor differentiation degree, microvessel density, and VEGF expression were also independent predictors of 5-year overall survival in the validation set (all *P* < 0.05). Moreover, the HR values for each factor showed consistent trends with those in the training set, confirming the stability and reliability of the core predictors in the model.

**Table 2:** Univariate and multivariate Cox regression analysis of factors associated with 5-year overall survival in ESCC patients.

Variable	Univariate Cox regression analysis		Multivariate Cox regression analysis	
	HR (95% CI)	*P*-value	HR (95% CI)	*P*-value
Age (per 1-year increase)	1.012(0.985-1.040)	0.386	1.008(0.975–1.042)	0.642
Gender (Male vs. Female)	1.214(0.781-1.888)	0.387	1.102(0.694–1.751)	0.681
Tumor diameter (per 1-cm increase)	1.302(1.023-1.658)	0.032	1.145(0.892–1.470)	0.287
AJCC stage (III vs. I/II)	2.478(1.564-3.926)	0.001	1.347(1.209–1.510)	0.010
Lymph node metastasis (Yes vs. No)	2.615(1.665-4.108)	0.001	2.915(1.982–4.288)	0.001
Tumor differentiation (High vs. Moderate-Low)	2.217(1.359-3.616)	0.001	1.235(1.105–1.276)	0.042
MVD (per 1-number increase)	1.068(1.023-1.115)	0.003	1.089(1.023–1.159)	0.007
VEGF expression (High vs. Low)	2.372(1.511-3.724)	0.001	2.451(1.127–6.372)	0.025
Preoperative weight loss (Yes vs. No)	1.988(1.273-3.104)	0.002	1.210(0.845–1.731)	0.298
Postoperative adjuvant therapy (Yes vs. No)	0.592(0.379-0.924)	0.021	0.745(0.512–1.085)	0.124

### Development of the nomogram prediction model

**Figure 2:**
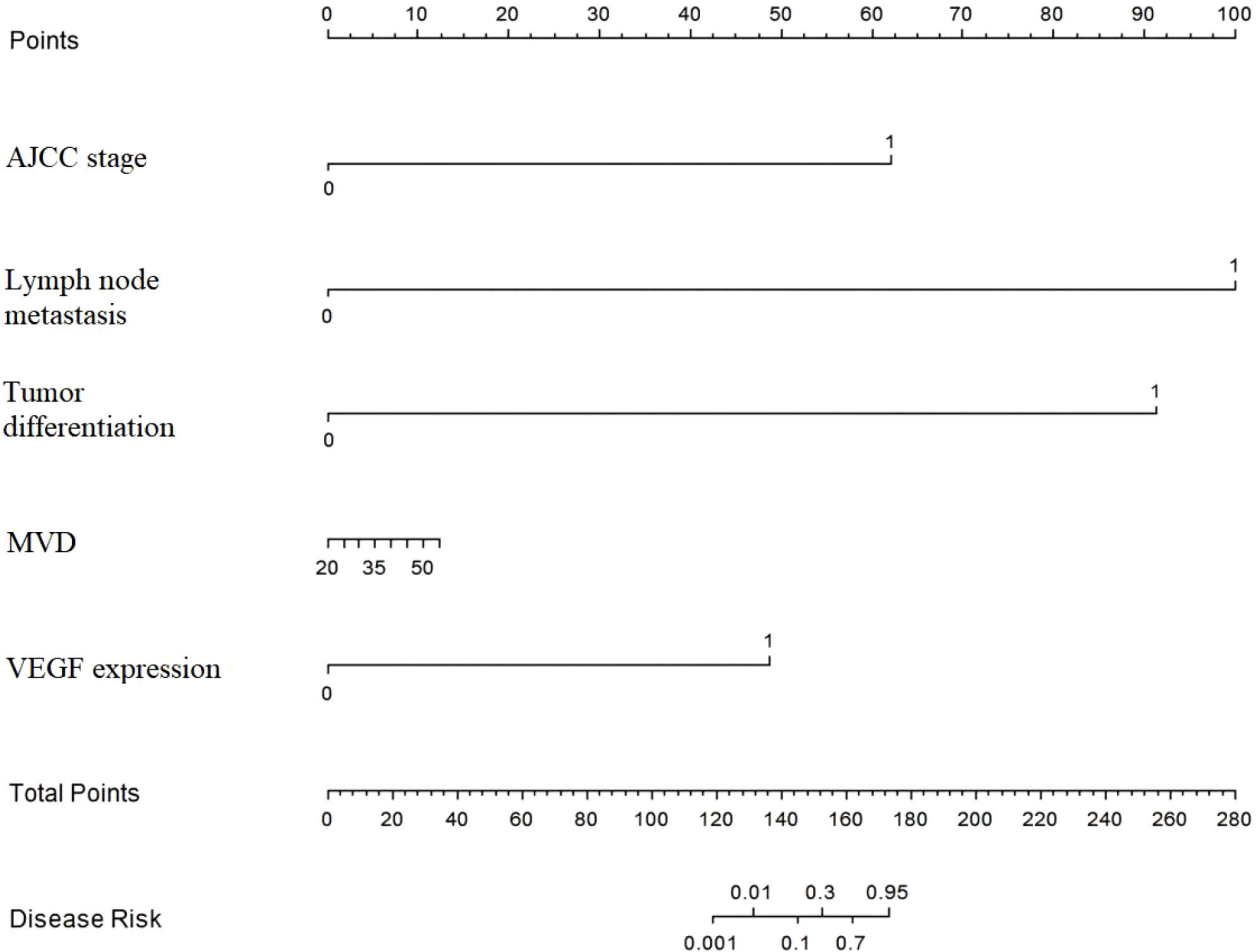
Nomogram model for predicting the 5-year post-operative survival rate of patients with esophageal squamous cell carcinoma. AJCC stage (I/II stage vs III stage); Lymph node metastasis (No vs Yes); Tumor differentiation (High differentiation vs Moderate-Low differentiation); MVD (per 1-number increase in HPF); VEGF expression (Low expression vs High expression). Usage method: First, find the corresponding score of each variable status of the patient on the score scale, sum all the scores to get the total score; then, find the corresponding 1-year, 3-year and 5-year OS probability of the total score on the disease risk scale, which is the predicted survival probability of the patient.

A nomogram model for predicting the 5-year post-operative survival rate of patients with esophageal squamous cell carcinoma was constructed based on the independent risk factors identified by multivariate Cox regression analysis. Corresponding score scales were assigned to each factor according to the regression coefficients of each factor, and the total score corresponded to the probability of prognostic death (**Figure 2**).

### Evaluation and validation of the nomogram prediction model

**Figure 3:**
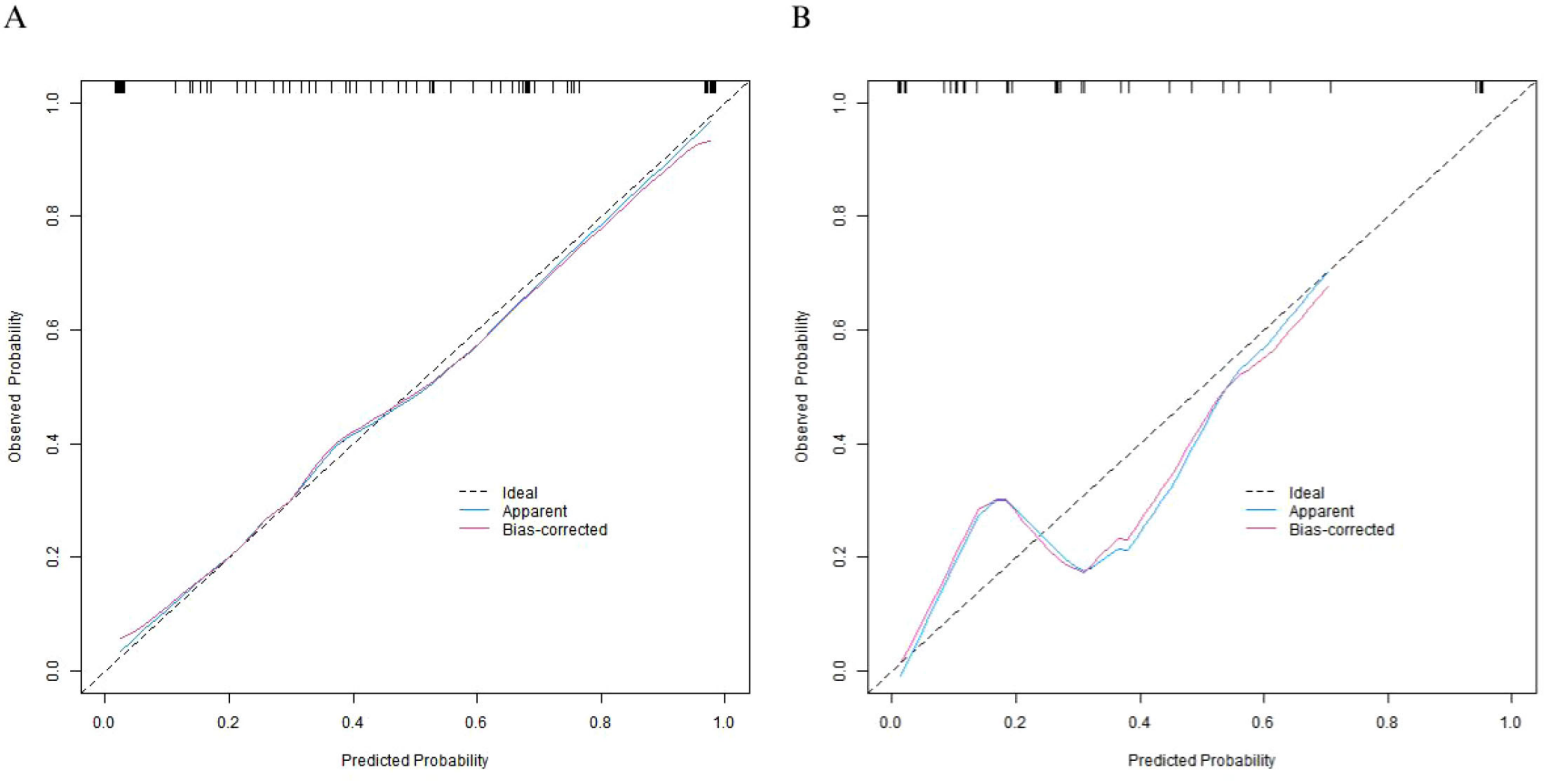
Calibration curves of the nomogram prediction model **(A)** the training set, **(B)** the validation set.

**Figure 4:**
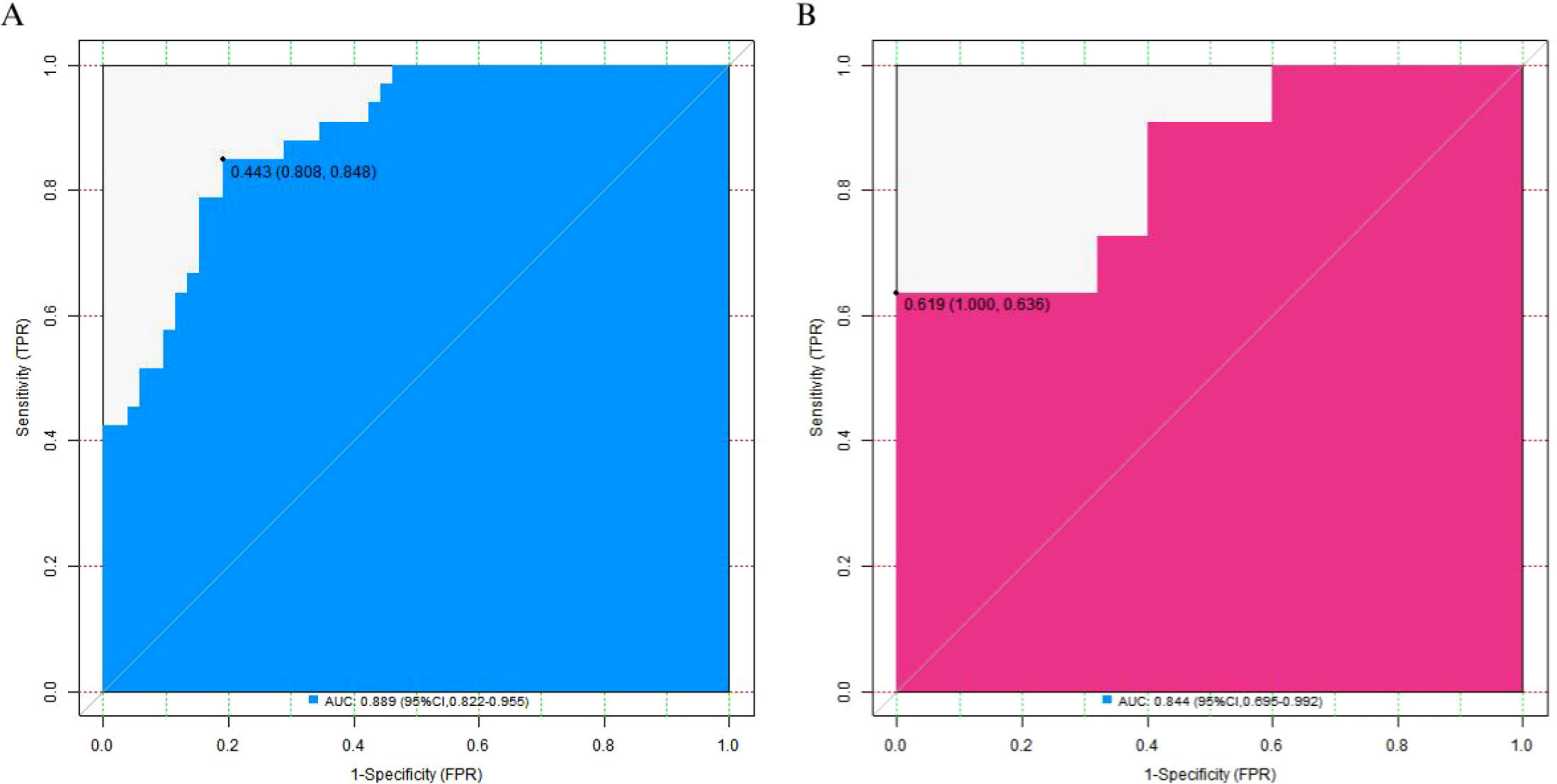
Receiver operating characteristic curves of the nomogram prediction model **(A)** the training set, **(B)** the validation set).

In the training set and the validation set, the C - index of the constructed nomogram prediction model was 0.886 and 0.845 respectively. Further analysis through calibration curves showed that there was good agreement between the predicted values of the model and the actual observed values, with the mean absolute errors being 0.129 and 0.145 respectively. Moreover, the results of the Hosmer - Lemeshow test indicated that the χ² values of the training set and the validation set were 7.226 (*P* = 0.513) and 14.077 (*P* = 0.080) respectively, as shown in **Figure 3**. In addition, ROC curve analysis showed the ability of the nomogram model to predict the 5-year postoperative survival rate of patients with esophageal squamous cell carcinoma. The AUC values of the training set and the validation set were 0.889 (95% CI: 0.822 - 0.955) and 0.844 (95% CI: 0.695 - 0.992) respectively, and the corresponding combinations of sensitivity and specificity were 0.848 and 0.808, as well as 0.727 and 0.600 respectively (**Figure 4**). We also tested a nomogram incorporating all variables that were significant in univariate analysis; its discrimination (C-index: 0.821 in training set, 0.793 in validation set) and calibration were inferior to those of the final model, confirming the benefit of selecting independent predictors via multivariable regression.

### Decision curve analysis of the nomogram prediction model

**Figure 5:**
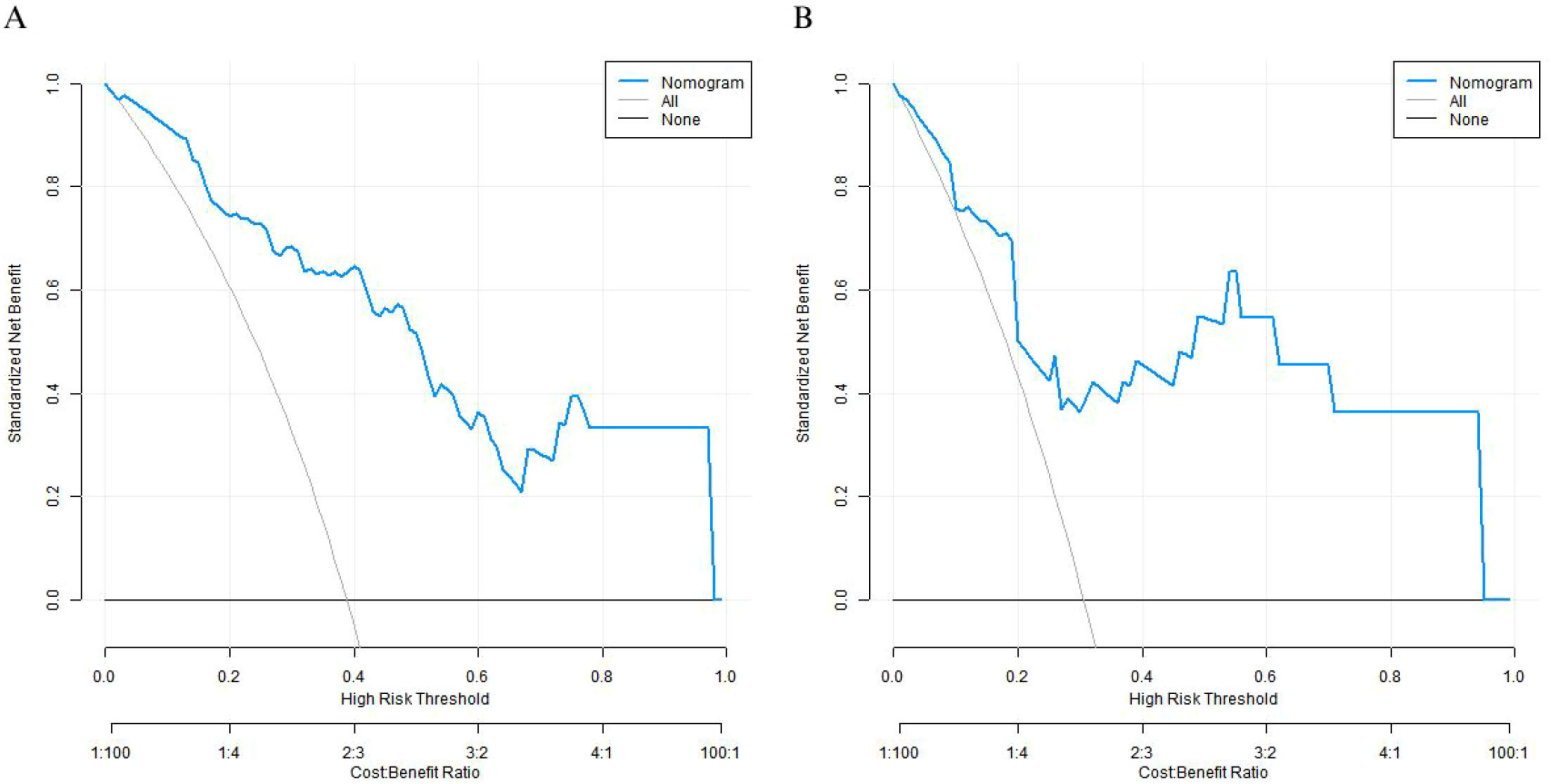
Decision curves of the nomogram prediction model **(A)** the training set, **(B)** the validation set.

Decision curve analysis indicated that when the threshold probability was between 0.2 and 0.8, the model was found to have a relatively high clinical net benefit in predicting the 5-year postoperative survival rate of patients with esophageal squamous cell carcinoma (**Figure 5****).**

## Discussion

Based on multivariate Cox regression analysis, this study successfully constructed and preliminarily validated a nomogram prediction model integrating AJCC stage, lymph node metastasis, tumor differentiation degree, microvessel density (MVD), and VEGF expression. A large number of nomogram models for ESCC postoperative survival prediction have been reported in previous studies, most of which only included traditional clinicopathological factors such as tumor stage, lymph node metastasis and differentiation degree, and a small number of studies incorporated a single molecular marker, while few integrated multiple key angiogenic molecular markers with clinicopathological factors. The nomogram demonstrated excellent discrimination and calibration in internal validation. Its core value lies in combining the classic anatomical prognostic framework with two key angiogenic molecular markers (MVD and VEGF), thereby achieving a more comprehensive and individualized assessment of postoperative survival risk for patients with esophageal squamous cell carcinoma compared with previous models. Its core value lies in combining the classic anatomical prognostic framework with key angiogenic molecular markers, thereby achieving a more comprehensive and individualized assessment of postoperative survival risk for patients with esophageal squamous cell carcinoma.

The traditional TNM staging system serves as the cornerstone of current clinical staging, with its value lying in the precise description of the local tumor invasion extent and metastatic status. However, this system primarily relies on morphological characteristics and struggles to fully explain the significant survival heterogeneity observed within patient groups at the same stage, indicating inherent limitations in its ability to characterize the intrinsic biological behavior of tumors (6).The core innovation and improvement of this study’s model lie in systematically integrating MVD and VEGF, two key angiogenic markers, to effectively address these shortcomings (8).From a mechanistic perspective, tumor angiogenesis is a critical step in tumor growth, invasion, and distant metastasis. MVD, by directly counting CD34-positive newly formed microvessels within tumor tissue, quantitatively reflects the tumor’s ability to induce angiogenesis and is considered the “gold standard” for assessing this process (9)VEGF, as the most potent pro-angiogenic factor, is the core regulatory signal that initiates and continuously drives this malignant process (10).Our multifactorial analysis confirmed that even after strict adjustment for traditional factors such as AJCC stage and lymph node status, MVD and VEGF remained independent predictors of postoperative survival. This finding strongly suggests that MVD and VEGF carry critical prognostic information, reflecting the biological aggressiveness of the tumor, which cannot be captured by traditional anatomical staging (11). Therefore, this model represents a significant paradigm evolution: moving from a singular “anatomical staging” towards an “integrated anatomical-biological functional assessment.” Its direct clinical significance is the ability to further stratify patients categorized within the same risk stratum by the traditional TNM system (e.g., all stage III) into subgroups with differing recurrence risks and survival probabilities based on the level of their tumor angiogenic activity (reflected by differences in MVD and VEGF expression) (12).This provides an objective, quantifiable tool for identifying “occult high-risk” patients. This study fills the gap of few ESCC prognostic models integrating multiple key angiogenic molecular markers with classic clinicopathological factors in previous studies, and its research results provide a new methodological basis for the precision prognostic assessment of ESCC, which is a meaningful advance in the existing literature (13).

To precisely quantify the incremental predictive value contributed by MVD and VEGF in future clinical translation, a key task is to conduct rigorous evaluation in large-scale, multi-center external validation cohorts using statistical methods such as the Net Reclassification Improvement (NRI) and Integrated Discrimination Improvement (IDI) indices (14).These metrics can objectively measure whether, and to what extent, the newly added biomarkers improve the model’s ability to correctly classify individual patient risk, thereby providing crucial decision-making evidence regarding the worthiness of incorporating these tests into routine clinical practice (15).Within a clear envisioned clinical translation pathway, a sufficiently externally validated model could be integrated into multidisciplinary team workflows as a reliable risk stratification tool to provide reference for postoperative adjuvant treatment decisions and the formulation of follow-up strategies, and its value in guiding individualized treatment needs to be further verified by prospective interventional clinical trials (16). For patients identified as high-risk by the model, clinicians could recommend more aggressive comprehensive treatment regimens based on this information, such as considering combining anti-angiogenic targeted therapy with standard adjuvant chemoradiotherapy or suggesting priority enrollment into relevant clinical trials (17). Simultaneously, a more intensive schedule for imaging and tumor marker surveillance could be planned (18). Conversely, for low-risk patients, the model could provide a basis for adopting a relatively conservative follow-up strategy or avoiding unnecessary intensive treatment, aiding in minimizing treatment-related toxicities, improving quality of life, and conserving healthcare resources while ensuring efficacy, fully aligning with the “risk-adapted” treatment philosophy advocated by precision oncology (19). The prerequisite for realizing this precision management loop, however, is promoting the standardization and reproducibility of MVD and VEGF detection techniques, such as adopting digital pathology image analysis systems to replace traditional manual interpretation, thereby minimizing inter-laboratory and inter-observer variability (20).

It is worth noting that manual MVD counting and VEGF immunohistochemical grading have certain inter-observer variability, which is a key factor that may affect the clinical reproducibility of this nomogram model. The inter-observer variability is mainly caused by the subjectivity of pathologists in identifying tumor microvessel hot-spot areas, judging the boundary of CD34-positive microvessels, and evaluating the staining intensity and positive rate of VEGF expression. Digital pathology and artificial intelligence-based automated scoring technology are effective ways to mitigate this problem. Digital pathology can realize the standardized digitization, storage and remote sharing of pathological slices, which avoids the differences in slice observation caused by traditional physical slice damage and uneven staining, and facilitates the unified interpretation and consultation of multiple pathologists. Automated scoring technology can quantitatively count the number of CD34-positive microvessels in the hot-spot area and calculate the positive cell rate and staining intensity of VEGF expression through image segmentation and feature extraction algorithms, which eliminates the subjectivity of manual visual judgment, significantly improves the consistency and reproducibility of MVD and VEGF detection results, and provides an important technical guarantee for the clinical translation and popularization of this nomogram model.

This study has the following limitations. Firstly, as a retrospective, single-center study, its conclusions may be subject to selection bias, and the limited sample size may restrict the model’s stability and generalizability in broader populations. Secondly, although bootstrap resampling was employed for internal validation, the model has yet to be validated in an independent external cohort from different medical centers, which is an indispensable step for assessing its real-world applicability (21).Thirdly, the model constructed in this study primarily focuses on pathological and angiogenic indicators and does not incorporate multidimensional information known to significantly impact prognosis, such as patient performance status, systemic inflammatory markers, circulating tumor DNA, and tumor immune microenvironment characteristics. Fourthly, this study only constructed and validated the prognostic prediction model, and did not conduct prospective interventional studies to verify the direct value of the model in guiding individualized treatment, so the application of the model in clinical treatment tailoring needs to be further explored and verified. Finally, the assessment of MVD and VEGF is based on immunohistochemistry. Although routine protocols were followed, interpretation still carries a degree of subjectivity, which affects the objectivity and comparability of the results to some extent. Based on these points, future research should prioritize conducting rigorous multi-center external validation (22).Our research team plans to launch a multi-center, prospective external validation study in the next step, in cooperation with multiple tumor hospitals in northern, central and southern China, to enroll ESCC patients with different demographic characteristics and clinical treatment backgrounds. We will verify the predictive performance and generalizability of this model in an independent multi-center cohort, and further optimize the weight of each predictor in the model according to the external validation results to improve its clinical applicability and popularization value. Building upon this, further exploration can be undertaken to integrate multi-omics data, such as genomic variations, transcriptomic features, proteomics, and metabolomics, to construct a more robust and dynamic prognostic prediction system. More importantly, well-designed prospective, interventional clinical trials are ultimately needed to verify whether personalized treatment strategies guided by this prediction model can translate into tangible improvements in patients’ long-term survival outcomes (23).

In summary, the integrated model constructed in this study provides a new tool for the postoperative individualized prognostic management of patients with esophageal squamous cell carcinoma. It not only consolidates the value of traditional staging systems but, more importantly, by incorporating angiogenic molecular markers, quantifies and integrates tumor biological behavior characteristics into the prognostic assessment framework. This represents a beneficial exploration in the evolution of this field from “stage-oriented” towards “biology-oriented” precision assessment. Although the definitive establishment of its ultimate clinical value awaits external validation and confirmation through prospective studies, this research undoubtedly lays a methodological foundation for subsequent efforts towards more precise risk stratification and treatment decision-making.

## Data Availability

The original contributions presented in the study are included in the article/**Supplementary Material**. Further inquiries can be directed to the corresponding authors.
