# Supplementary material for: Construction and verification of 5-year survival prediction model for post-op ESCC patients
Source: Front Oncol. 2026 Apr 29;16:1798175. doi: 10.3389/fonc.2026.1798175 (PMC13167563; doi:10.3389/fonc.2026.1798175)
Supplement: Supplementary file 1 [file Table1.docx]

### Supplementary Table 1. Variable assignments for Cox regression analysis

| **Variable** | **Variable type** | **Assignment** |
| --- | --- | --- |
| Age | Continuous | Per 1‑year increase |
| Gender | Binary | Male = 1, Female = 0 (reference: Female) |
| Tumor diameter | Continuous | Per 1‑cm increase |
| AJCC stage | Binary | Stage III = 1, Stage I/II = 0 (reference: I/II) |
| Lymph node metastasis | Binary | Yes = 1, No = 0 (reference: No) |
| Tumor differentiation grade | Binary | High = 1, Moderate‑Low = 0 (reference: Moderate‑Low) |
| MVD | Continuous | Per 1‑number increase in HPF |
| VEGF expression | Binary | High expression = 1, Low expression = 0 (reference: Low expression) |
| Preoperative weight loss | Binary | Yes = 1, No = 0 (reference: No) |
| Postoperative adjuvant therapy | Binary | Yes = 1, No = 0 (reference: No) |
